# Identification of physiological clusters in acute hypoxemic respiratory failure patients undergoing non-invasive respiratory support using EIT-based t-SNE and spectral clustering

**DOI:** 10.1186/s40635-026-00912-6

**Published:** 2026-05-28

**Authors:** Gaetano Scaramuzzo, Valentina Bellini, Matteo Trevisani, Francesca Cinquegrana, Marta Bonanni, Marta Ciniero, Matteo Riccardo, Pierluigi Ferrara, Alessandro Trentini, Tiziana Bellini, Giulia Tini, Sara Uboldi, Danila Azzolina, Savino Spadaro, Carlo Alberto Volta, Elena Giovanna Bignami

**Affiliations:** 1 Department of Translational Medicine, University of Ferrara, Via Aldo Moro, 8, 44121 Ferrara, Italy; 2 Department of Emergency, Azienda Ospedaliera-Universitaria Di Ferrara, Ferrara, Italy; 3 Anesthesiology, Critical Care and Pain Medicine Division, Department of Medicine and Surgery, University of Parma, Viale Gramsci 14, 43126 Parma, Italy; 4 Department of Neuroscience and Rehabilitation, University of Ferrara, Via Luigi Borsari 46, 44121 Ferrara, Italy; 5 Intellico Srl, Milan, Italy; 6 Department of Translational Medicine, University of Naples, Naples, Italy

**Keywords:** Acute hypoxemic respiratory failure, Electrical impedance tomography, Non-invasive respiratory support, Unsupervised clustering, Pendelluft, Intubation risk

## Abstract

**Purpose:**

Identifying physiological clusters in acute hypoxemic respiratory failure (AHRF) may help to personalize non-invasive respiratory support (NIRS). Electrical impedance tomography (EIT) provides real-time, regional information on tidal ventilation, but its value for clustering AHRF patients undergoing NIRS has not been established.

**Methods:**

We conducted a single-center observational study including adults with AHRF monitored with EIT during NIRS. Tidal ventilation images were pre-processed, normalized, and embedded into a 2-dimensional space using t-SNE. Spectral clustering was applied to identify distinct imaging patterns. Clinical, physiological and laboratory variables were compared across clusters. The association between cluster membership and intubation at 7 days was assessed using penalized Cox regression adjusted for age, BMI, PaCO₂ and ROX index.

**Results:**

Thirty-two patients were enrolled. Spectral clustering identified three distinct clusters of tidal images. Clusters differed in clinical severity and physiological profile: Cluster 1 was characterized by shorter stature and higher SAPS II; Cluster 2 showed the highest pendelluft; Cluster 3 exhibited symmetric ventilation with low pendelluft. These phenotypes also differed in hemodynamics, including heart rate and shock index. Cluster membership was independently associated with intubation at 7 days. Compared with Cluster 3, both Cluster 1 and Cluster 2 showed a significantly lower hazard of intubation (HR 0.115, *p* = 0.017 and 0.042, *p* = 0.002, respectively).

**Conclusions:**

Unsupervised clustering of EIT tidal images is feasible in AHRF and identifies distinct physiological clusters with different short-term outcomes. These findings support the potential role of EIT-based imaging patterns for early stratification of patients undergoing NIRS.

**Supplementary Information:**

The online version contains supplementary material available at 10.1186/s40635-026-00912-6.

## Introduction

Non-invasive respiratory support (NIRS) is widely used in the intensive care unit (ICU) for a variety of clinical indications [1–5]. However, its role in patients with acute hypoxemic respiratory failure (AHRF) remains controversial. Although NIRS can improve gas exchange and reduce inspiratory muscle effort [6–8], its impact on clinically meaningful outcomes is still uncertain. Current recommendations increasingly emphasize the need for personalized respiratory support strategies, recognizing the substantial heterogeneity underlying AHRF. In this context, identifying disease phenotypes that may respond differently to NIRS could help optimize treatment titration and potentially improve patient outcomes.

Previous efforts to phenotype acute respiratory failure have primarily focused on acute respiratory distress syndrome (ARDS). Imaging-based approaches using computed tomography have identified distinct morphological patterns, such as focal and diffuse ARDS, that differ in their response to positive end-expiratory pressure (PEEP) titration [9], although these associations with overall survival have not been consistent. Recently, biomarker-based classifications have described hyperinflammatory and hypoinflammatory phenotypes, suggesting differential responses to ventilatory and pharmacological interventions [10, 11]. Despite their conceptual relevance, the clinical utility of these phenotypes remains limited, largely due to the heterogeneity of AHRF/ARDS and the challenges associated with bedside implementation [12].

Electrical impedance tomography (EIT) is a non-invasive bedside monitoring technique that measures regional impedance changes during tidal breathing using a thoracic electrode belt, allowing real-time visualization of regional ventilation distribution [13, 14]. EIT has been successfully applied in perioperative and mechanically ventilated patients to characterize ventilation patterns and identify physiological phenotypes [15, 16]. However, its application for characterizing patients with AHRF receiving non-invasive respiratory support remains largely unexplored.

Recently, artificial intelligence (AI)-driven approaches have emerged as promising tools to address the complexity and heterogeneity of acute respiratory failure. Unsupervised machine-learning methods enable data-driven identification of latent physiological patterns without reliance on predefined labels or outcomes, making them especially suitable for exploratory phenotyping in critical care without relying on pre-existing data or measured features. When combined with high-resolution bedside monitoring technologies, such approaches offer the potential to support individualized respiratory management strategies and improve early risk stratification [17]. Unsupervised clustering is a data-driven statistical approach that identifies groups of similar observations within a dataset without relying on predefined labels or outcomes, in contrast to supervised machine-learning methods. In medical research, this approach is particularly valuable in heterogeneous populations, where it can uncover latent structures and previously unrecognized disease subtypes [18]. When applied to imaging-derived features, unsupervised clustering may support precision medicine by improving patient stratification and guiding individualized therapeutic strategies.

In this context, this EIT-based data-driven approach combining t-SNE and spectral clustering could represent a bedside tool to identify distinct physiological clusters among patients with AHRF undergoing NIRS, potentially associated with different clinical outcome trajectories. The primary aim of this study was, therefore, to assess the feasibility of applying an unsupervised clustering approach to EIT tidal ventilation images to identify physiological clusters in patients with AHRF receiving non-invasive respiratory support. Secondary aims were to evaluate clinical differences among the identified clusters and to explore their association with patient-centered outcomes and prognostic risk for intubation.

## Methods

### Study population

**Fig. 1:**
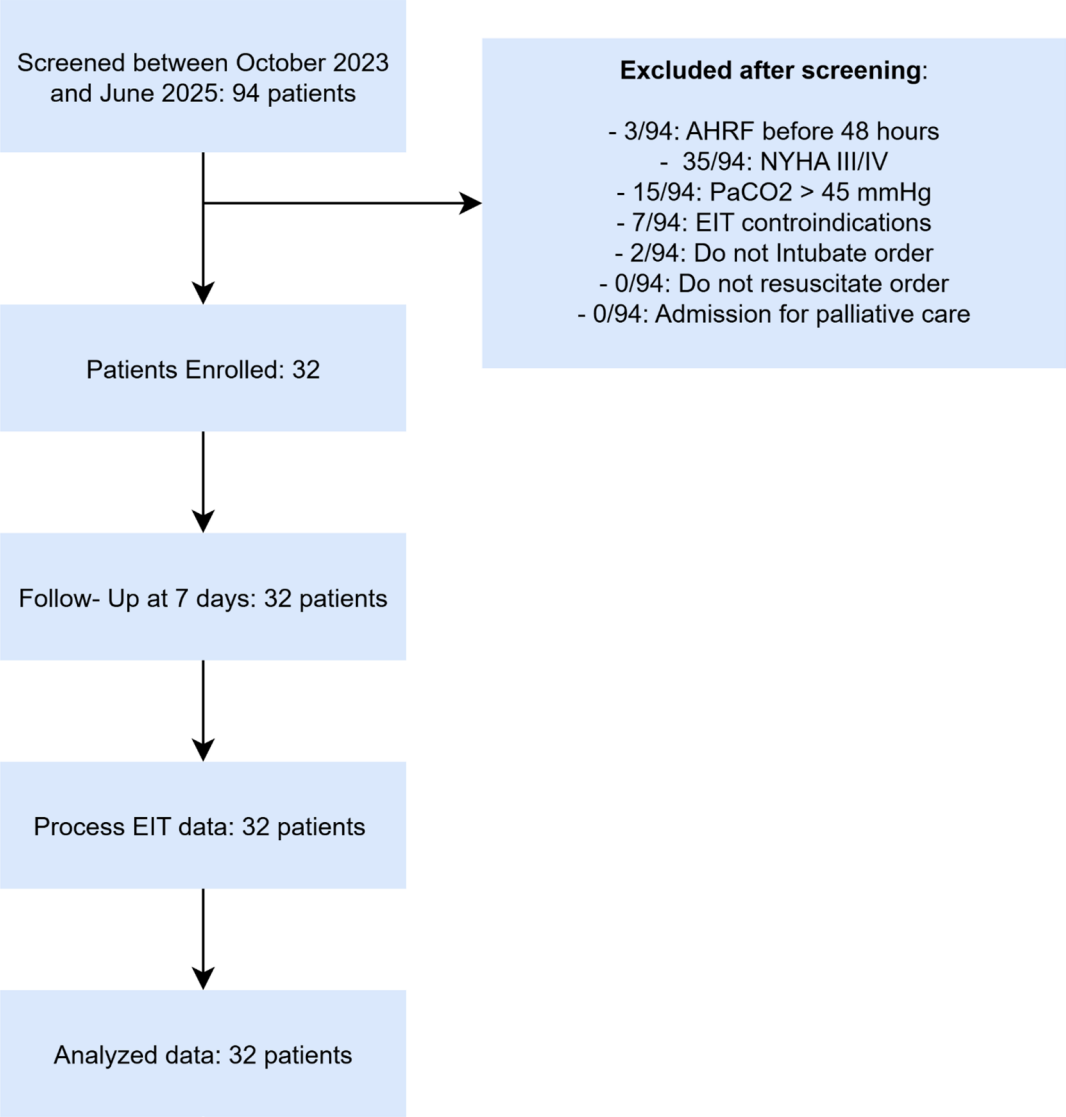
Flow diagram detailing the screening, inclusion, and exclusion of patients enrolled in the study. The figure illustrates the sequential steps used to derive the final analytical cohort, including initial assessment, application of eligibility criteria, and assignment to clusters

This prospective observational study was conducted in the Intensive Care Unit of the Azienda Ospedaliero-Universitaria di Ferrara between October 2023 and June 2025. Adult patients (≥18 years) were enrolled within 48 h from the diagnosis of acute hypoxemic respiratory failure (AHRF) and NIRS start, provided they were monitored with electrical impedance tomography (EIT) and invasive arterial blood pressure monitoring. Exclusion criteria were contraindications to EIT (e.g., pacemaker or implantable cardioverter-defibrillator, pregnancy), chronic respiratory failure (home oxygen therapy) and advanced chronic lung disease (COPD GOLD 3 and 4 stage or severe/very severe asthma) or acute-on-chronic lung disease (acute asthma attack or COPD-exacerbation), severe cardiac insufficiency (NYHA class III–IV), hypercapnia (PaCO₂ > 45 mmHg), do-not-intubate or do-not-resuscitate orders, and admission for palliative care. The study was approved by the Ethics committee of Area Vasta Centro on the 14/09/2023 with the code CE‐AVEC 598‐2023‐Oss‐AOUFe. A flowchart of the study is reported in Fig. 1.

### Data collection

Clinical data were collected at enrollment, defined as within 48 h from the diagnosis of AHRF. Clinical outcomes, i.e., intubation rate and mortality, were assessed at 7 and 28 days after enrollment.

### Clinical data

Demographic, clinical, and laboratory data were collected at the time of enrollment. Demographic variables included age, sex, height, weight, date of hospital admission, date of ICU admission, admission diagnosis, comorbidities, Sequential Organ Failure Assessment (SOFA) score, Simplified Acute Physiology Score II (SAPS II), Murray score, ROX index (defined as the ratio between SpO₂/FiO₂ and respiratory rate, as previously described [19, 20]), HACOR score [21] and smoking status.

Physiological and clinical parameters recorded at enrollment included respiratory rate (breaths/min), heart rate (beats/min), peripheral oxygen saturation (SpO_2_, %), arterial blood pressure (mmHg), fraction of inspired oxygen (FiO₂), type of ventilatory support, ventilator parameter settings and ongoing pharmacological therapies. Furthermore, the shock index was calculated as the ratio between heart rate and systolic blood pressure [22].

Arterial blood gas analysis was performed at enrollment, including pH, partial pressures of oxygen and carbon dioxide (PaO₂ and PaCO₂, mmHg), arterial lactate concentration (mmol/L), and bicarbonate concentration (mmol/L). Laboratory variables collected included hemoglobin (g/dL), white blood cell count (cells/mm^3^), platelet count (cells/mm^3^), creatinine (mg/dL), bilirubin (mg/dL), C-reactive protein (mg/dL), and procalcitonin (ng/mL).

### EIT data processing

At enrollment, EIT data were acquired using a dedicated EIT monitoring system (Pulmovista 600, Drager®, Lubeck, Germany). EIT electrode placement and signal acquisition were performed according to previously described methods [14]. Briefly, a 16-electrode belt was positioned at the fourth–fifth intercostal space, and the ICU mattress was set to static mode. Spontaneous breathing was recorded for at least 2 min at study entry. Raw EIT tidal images (.eit format) were exported and converted into binary format using proprietary software. Subsequent analyses were performed using a custom-made MATLAB script. For each patient, after recognizing major artifacts (cough, patient movement, abnormal breath), we selected 5 non-consecutive and representative acts in stable EELI periods. On these acts the following EIT-derived variables were computed as mean values:

- Pendelluft, calculated using the impedance-based method proposed by Menga et al. [7];
- Regional ventilation distribution across four regions of interest (ROI 1: most ventral to ROI 4: most dorsal, defined as fixed regions of 8 pixel rows each);
- Center of ventilation (CoV), defined as the geometric mean of the tidal impedance variation and characterized by two variables, x (horizontal axis, 0 = right; 32 = left) and y (vertical axis, 0 = dorsal, 32 = ventral) [23];
- Global inhomogeneity index, defined as the inhomogeneity among different pixels’ relative impedance variation [23];
- Dorsal fraction of ventilation, DFV, defined as the percentage of tidal volume received by the dorsal part of the lung (ROI3 + ROI4) [23].

Complete formulas for EIT-derived parameters’ calculation are reported in the Online supplement.

### Tidal images analysis and clustering

To facilitate clustering analysis, pixel values from EIT images were normalized to the [0,1] range. Image matrices (1024 dimensions) were then reduced to a two-dimensional space using t-distributed stochastic neighbor embedding (t-SNE) [24] with cosine distance as the similarity metric (see online supplement for formulas). To reduce noise and speed up computation, we first performed an initial reduction to 50 components with principal component analysis (PCA); due to the small dimension of the dataset we set the perplexity t-SNE parameter to a level of 5, as suggested in the Python scikit-learn library. To ensure results’ reproducibility, we set the “random_state” parameter to 42. The resulting two-dimensional representation allowed visualization and clustering of tidal images based on similarity. Spectral clustering was applied using an affinity matrix constructed from nearest-neighbor graphs [25] “random_state” set to 0 for reproducibility.

**Fig. 2:**
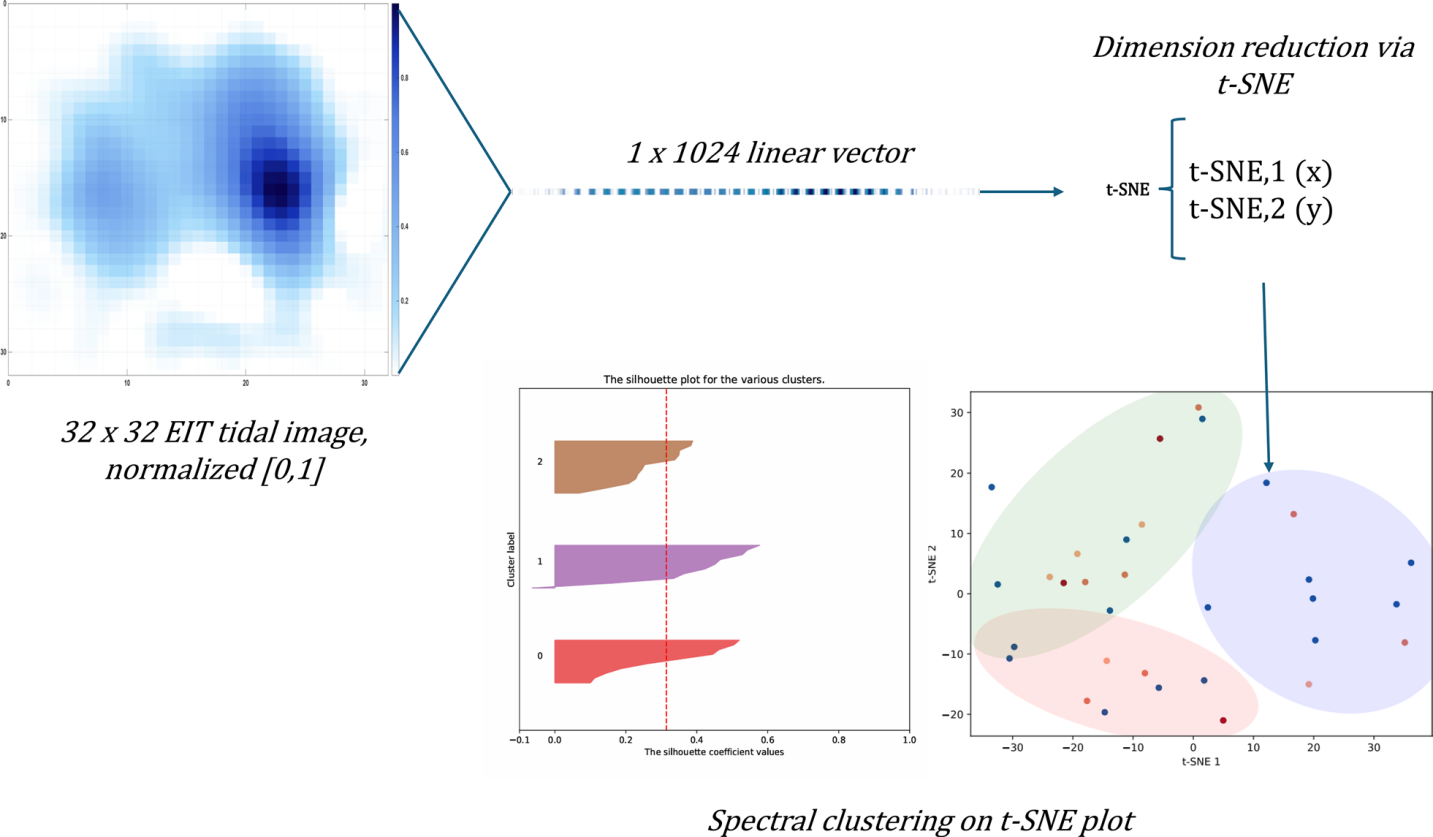
Clusters production from raw EIT tidal images. EIT images are first normalized between [0 1] to standardize intensity information. The t-SNE algorithm is then applied to embed the images in a two-dimensional feature space, preserving local similarity structure. Spectral clustering is subsequently used on the resulting embedding to identify groups of EIT images with similar characteristics

To select the appropriate number of clusters, we performed spectral clustering with k ranging from 2 to 5. For each k, we computed the silhouette score [26], the proportion of samples with negative silhouette coefficients, and inspected cluster size distribution to assess balance. To assess clustering stability, we repeated the analysis 10 times with different random initialization seeds and computed the Jaccard index among cluster label assignments. A graphical representation of the image processing process can be found in Fig. 2.

### Statistical analysis

Given the exploratory nature of the study, sample size estimation was based on feasibility and consistency with similar studies on advanced respiratory mechanics. A convenience sample of at least 30 patients was considered appropriate for exploratory outcome assessment [27].

Normality was assessed using the Shapiro–Wilk test. Normally distributed continuous variables are presented as mean ± standard deviation, while non-normally distributed variables are reported as median (interquartile range, [25th–75th] percentile). Categorical variables are presented as numbers and percentages. Differences among clusters were evaluated using one-way analysis of variance (ANOVA) for normally distributed continuous variables and the Kruskal–Wallis test for non-normally distributed continuous variables. Categorical variables were compared using Pearson’s Chi-square test.

Owing to the limited sample size and the small number of events, a penalized Cox proportional hazards model was used to evaluate the association between EIT-based cluster membership and intubation within 7 days. Penalization was adopted to reduce overfitting and stabilize regression estimates in the presence of sparse data. Results are reported as hazard ratios with 95% confidence intervals and should be interpreted as exploratory and prognostic rather than causal. MATLAB R2022a was used to process EIT data and extract features. R software (version 4.2.1) was used for clustering procedures and statistical analyses. The *NbClust* package was used for internal validation and to determine the optimal number of clusters.

## Results

### Baseline characteristics of the population

We enrolled 32 patients, including 13 (41%) females and 19 (59%) males, with a median age of 72 years and a median BMI of 27.2 kg/m^2^. The most frequent concomitant admission diagnoses were hypovolemic shock and septic shock (14/32, 43.8%). Median SAPS II at admission was 43 [32–53.5], SOFA score was 3 [2–5], and Murray score was 2 [1.5–2.5]. The most frequent comorbidities were cardiovascular and respiratory comorbidities, respectively, present in 23/32 (72%) and 9/32 (28%) patients. Patients who failed NIRS underwent invasive mechanical ventilation after a median time of 2.5 [2–4] days, and the duration of invasive mechanical ventilation was 9 [6–11] days. Other baseline characteristics of the population are described in Table 1. The study flowchart is shown in Fig. 1.

**Table 1:** Demographic features of the population

	Overall (*n* = 32)
Age (years)	71.5 [62–77]
Sex, females, *n* (%)	13 (41%)
Height (cm)	169 [160–175]
BMI (kg/m^2^)	27.2 [24–30.75]
SOFA score	3 [2–5]
SAPS II	43 [32–53.5]
ROX index	8.9 [6.4–10.5]
Murray score	2 [1.5–2.5]
Total fluid balance (ml/kg)	21.1 [3–69]
Comorbidities	
Chronic kidney disease	5 (15.6%)
Respiratory disease	9 (28.1%)
Neurological disease	5 (15.6%)
Cardiovascular disease	23 (71.9%)
Metabolic disease	13 (40.6%)
Non-smokers	26 (81.3%)
Ex-smokers	4 (12.5%)
Current smoker	2 (6.3%)
Admission diagnosis	
Pneumonia	5 (15.6%)
Shock	14 (43.8%)
Neuromuscular diagnosis	4 (12.5%)
Post-surgery admission	8 (25%)
Trauma	1 (3.1%)
Respiratory support modality at enrollment	
NIV	9 (28.1%)
HFNC	23 (71.9%)

BMI: body mass index; SOFA: Sequential Organ Failure Assessment; SAPS II: Simplified Acute Physiology Score II; NIV: non-invasive ventilation; HFNC: high-flow nasal cannula

### t-SNE and spectral clustering analysis

**Fig. 3:**
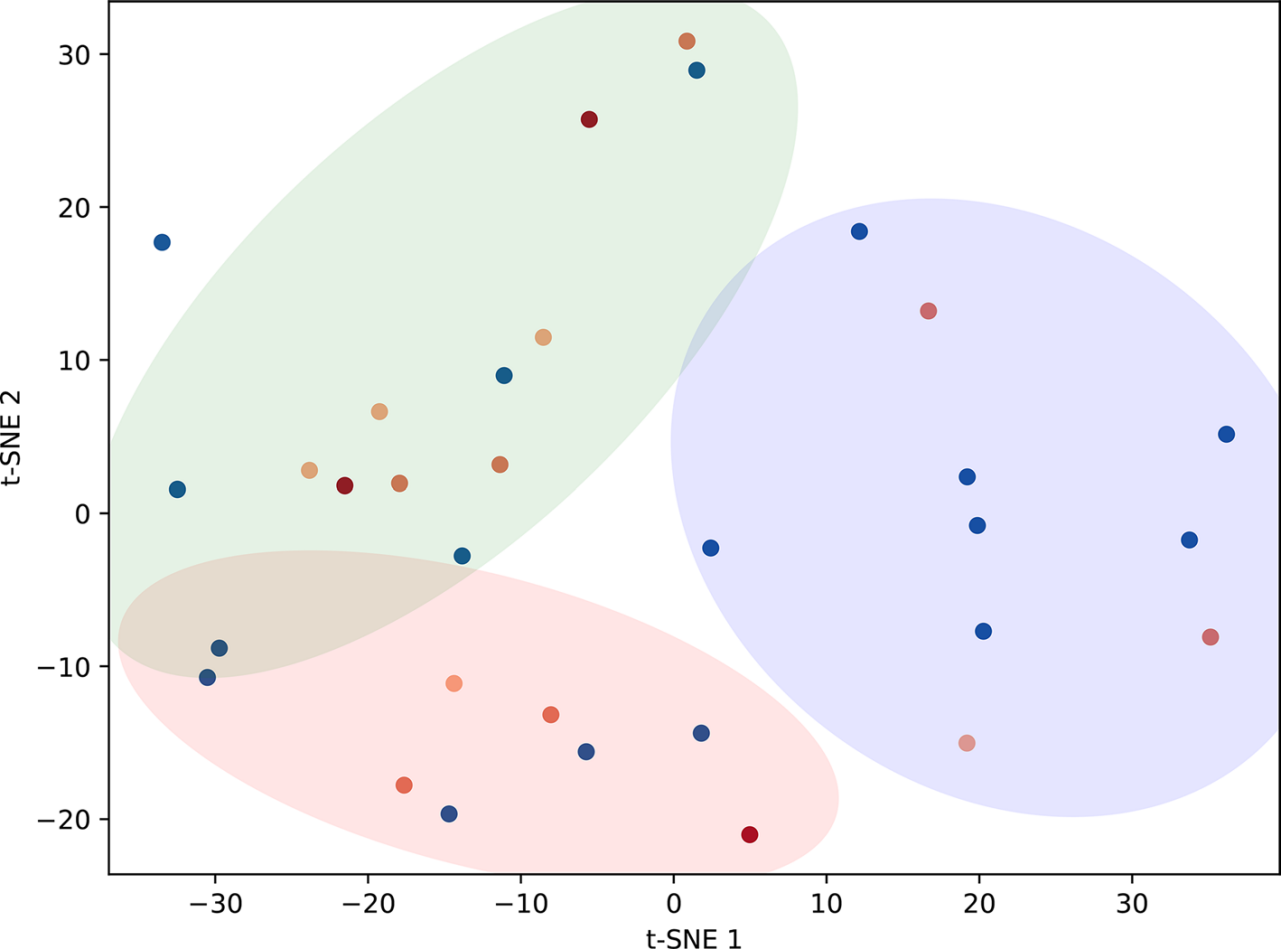
Results of t-SNE plus spectral clustering of EIT images. Two-dimensional t-distributed stochastic neighbor embedding (t-SNE) representation of the study population, colored according to the three clusters identified by the spectral clustering algorithm. Each point corresponds to an individual patient projected onto the two new axes generated by t-SNE (t-SNE 1 and t-SNE 2). Blue dots = NIRS success at seven days, alive at 28 days; light orange dots = NIRS failure at seven days, alive at 28 days; orange dots = NIRS success at seven days, dead at 28 days; red dots = NIRS failure at seven days, dead at 28 days. C1 = red cluster; C2 = blue cluster; C3 = green cluster

Based on silhouette score analysis, the best number of clusters describing our data was 3 (Fig. 3). Jaccard index among cluster label assignments provided a mean value of 1.0, indicating robust results. Two clusters are composed of 10 patients each, while 1 contains 12 patients. In the two-dimensional embedding space, the cluster located in the lower-left quadrant was labeled cluster 1 (C1), the one in the right was labeled cluster 2 (C2) and the cluster in the upper-left quadrant was labeled cluster 3 (C3).

### Demographics and clinical characteristics of clusters

Differences in demographic characteristics among clusters are summarized in Table 2. At admission, clusters differed significantly in height (online supplementary Figure S1) and SAPS II score (Figure S2).

**Table 2:** Demographic features among the different EIT-based clusters

	Cluster I (*n* = 10)	Cluster II (*n* = 10)	Cluster III (*n* = 12)	*p* value
Age (years)	71 [66–77]	74 [69–80]	65 [57.5–75]	0.204
Sex, females	5(50%)	3 (30%)	4 (33%)	0.608
Height (cm)	164.5 [160–168]	170 [162.25–177.25]	171.5 [166.75–178.5]	**0.023**
BMI (kg/m2)	27.3 [23.9–31.1]	27.1 [25.5–31.1]	25.5 [23.8–28.35]	0.715
SOFA score	4.5 [2–6]	2.5 [2–5]	3[2–4.5]	0.555
SAPS II at ICU entrance	50 [45–67]	32.5 [25–43]	42.50 [37.00–49.75]	**0.021**
ROX index at enrollment	10.2 [5.8–11.1]	8.6 [6.7–10.3]	8.9 [6.9–9.9]	0.939
Standard HACOR score at enrollment	2.5 [0.5–4]	5 [3–5]	3 [1.5–5.25]	0.473
Murray Score	1.5 [1–1.5]	2.3 [1.5–3]	2 [1.5–2.5]	0.211
Total fluid balance (ml/kg)	38.46 [17.59–81.99]	4.88 [-0.60–21.11]	37.76 [4.70–138.71]	0.198
Comorbidities				
Chronic kidney disease	1 (10%)	3 (30%)	1 (8.3%)	0.318
Respiratory disease	4 (40%)	4 (40%)	1 (8.3%)	0.156
Neurological disease	3 (30%)	2 (20%)	0 (0%)	0.140
Cardiovascular disease	9 (90%)	8 (80%)	6 (50%)	0.091
Metabolic disease	4 (40%)	5 (50%)	4 (33%)	0.73
Non-smokers	8 (80%)	6 (60%)	12 (100%)	0.093
Ex-smokers	1 (10%)	3 (30%)	0 (0%)	
Smoker	1 (10%)	1 (10%)	0 (0%)	
Admission diagnosis				
Pneumonia	2 (20%)	0 (0%)	3 (25%)	0.247
Shock	5 (50%)	5 (50%)	4 (33%)	0.655
Neuromuscular diagnosis	2 (20%)	2 (20%)	0 (0%)	0.254
Post-surgery admission	1 (10%)	2 (20%)	5(41%)	0.211
Trauma	0 (0%)	1 (10%)	0(0%)	0.321
Respiratory support modality at enrollment				
NIV	3 (30%)	2 (20%)	4 (33.3%)	0.777
HFNC	7 (70%)	8 (80%)	8 (67%)	
PS, cmH_2_O	8 [8–12]	7 [6.5–7.5]	9 [7.5–10]	0.42
PEEP, cmH_2_O	8 [7, 8]	8 [8–8]	7 [5.5–8]	0.45
Flow, liter/min (HFNC)	60 [60–60]	60 [50–60]	60 [60–60]	0.67
FiO2, % (NIV)	50 [46–55]	45 [42–47]	45 [41–58]	0.84
FiO2, % (HFNC)	54 [44–58]	60 [50–65]	50 [47–59]	0.57

BMI: body mass index; SOFA: Sequential Organ Failure Assessment; SAPS II: Simplified Acute Physiology Score II; NIV: non-invasive ventilation; HFNC: high-flow nasal cannula. One-way ANOVA/Kruskal–Wallis test according to distribution

Patients in C1 were significantly shorter than those in C2 and C3 (164.5 [160–168] vs 170 [162.5–177.25] vs 171.5 [166.75–178.5] cm, *p* = 0.023). Although C1 included a higher proportion of female patients (5 [50%] vs 3 [30%] vs 4 [33%]), gender distribution did not differ significantly among clusters (*p* = 0.608). C1 also exhibited higher disease severity, with significantly higher SAPS II scores compared with the other clusters (50 [45–67] vs 32.5 [25–43] vs 42.5 [37 –49.75], *p* = 0.021).

Regarding clinical parameters, heart rate differed significantly among clusters, with higher values observed in C3 (83 [74–110] vs 70 [50–85] vs 94 [73.5–109] bpm, *p* = 0.018). The hemodynamic difference among clusters was further reflected by the difference in the shock index, which was significantly higher in C2 (1.34 [0.99–1.87] vs 1.95 [1.56–2.62] vs 1.45 [1.25–1.79], *p* = 0.009, figure S2). No other differences were found in hemodynamic, respiratory, and laboratory variables (Tables 3 and 4). Finally, the ratio of patients undergoing HFNC and NIV was comparable among clusters, as well as their ventilatory settings (Table 2).

**Table 3:** Clinical features of the study population and by clusters

	Cluster I (*n* = 10)	Cluster II (*n* = 10)	Cluster III (*n* = 12)	Overall (*n* = 32)	*p* value
Mean arterial pressure (mmHg)	89.2 [70–93.3]	86.3 [83.3–91.7]	86.6 [82.2–95.8]	86.6 [80.6–93.3]	0.647
Systolic arterial pressure (mmHg)	117 [101–136]	130.5 [126–138]	136 [120–140]	130 [119–140]	0.309
Heart rate (bpm)	83 [74–110]	70 [50–85]	94 [73.5–109]	80.5 [70–97.5]	**0.018**
Shock index	1.34 [0.99–1.87]	1.95 [1.56–2.62]	1.45 [1.25–1.79]	1.54 [1.24–1.93]	**0.009**
Arterial oxygen saturation (%)	97 [95–98]	97.5 [93–99]	97.5[96–98.5]	97 [95–99]	0.922
Respiratory rate (breaths/min)	20.5 [17–30]	20 [18–25]	22.5 [17–28]	20 [17–28]	0.914
FiO2 (%)	53.5 [44.7–59]	52.5 [50–65]	50 [44–58.7]	50 [44.5–60]	0.691
PaO2/FiO2 (mmHg)	185 [130–255]	157.5 [101–200]	173 [120–222]	171 [120.5–225]	0.238
GCS at enrollment	15 [15–15]	15 [15–15]	15 [15–15]	15 [15–15]	0.57
Adjunctive therapy					
Sedation, yes, *N* (%)	6 (60%)	4 (40%)	10 (83%)	20 (62.5%)	0.11
Corticosteroids, yes, *N* (%)	5 (50%)	2 (20%)	5 (50%)	12 (37.5%)	0.357
Vasoactive, yes, *N* (%)	3 (30%)	1 (10%)	2 (20%)	6 (18.75%)	0.505
Acute antihypertensive use, yes, *N* (%)	4 (40%)	4 (40%)	8 (80%)	16 (50%)	0.344

FiO2: fraction of inspired oxygen; PaO2: oxygen arterial partial pressure. One-way ANOVA/Kruskal–Wallis/Chi-square test according to data type and distribution

**Table 4:** Laboratory findings in the different EIT clusters and in the global population

	Cluster I (*n* = 10)	Cluster II (*n* = 10)	Cluster III (*n* = 12)	Overall (*n* = 32)	*p* value
pH	7.45 [7.43–7.52]	7.45 [7.41–7.48]	7.46 [7.40–7.49]	7.46 [7.41–7.49]	0.942
PaO_2_ (mmHg)	92.2 [75.8–101]	76.2 [63–128]	87.95 [73.7–100]	88.6 [70.7–101]	0.895
PaCO_2_ (mmHg)	42.05 [38.2–46]	43.7 [42–57]	39.8 [37.6–43.9]	42.2 [38.4–45.45]	0.16
Arterial lactates (mmol/L)	0.9 [0.7–1.2]	0.85 [0.8–1.1]	1.05 [0.85–1.4]	1 [0.7–1.25]	0.664
Bicarbonates (mmol/L)	29.35 [23.5–34.6]	31.2 [29.1–35]	30.55 [24.45–32]	29.95 [27.1–33.2]	0.186
Hemoglobin (g/dL)	9 [9–11]	9.5 [8–11]	9.5 [8–12.5]	9 [8–11.5]	0.827
Leukocytes (× 10^3^/mm^3^)	10.8 [7.3–15.4]	14.7 [12.5–23]	16.6 [10.9–24.6]	14.3 [9.8–21.4]	0.105
Creatinine (mg/dL)	0.97 [0.78–1.19]	1.53 [0.71–2.16]	1.09 [0.73–1.6]	1 [0.74–1.53]	0.739
C-reactive protein (mg/dL)	11.5 [9–20.2]	10.9 [7–15]	16.3 [5–20.6]	12.41 [6.95–20]	0.876
Procalcitonin (ng/mL)	2 [0–6]	2.2 [0.07–3.4]	1 [0.2–2.5]	1.13 [0.07–3.84]	0.788

PaO2: arterial oxygen partial pressure; PaCO2: arterial carbon dioxide partial pressure

### EIT-derived parameters among clusters

**Fig. 4:**
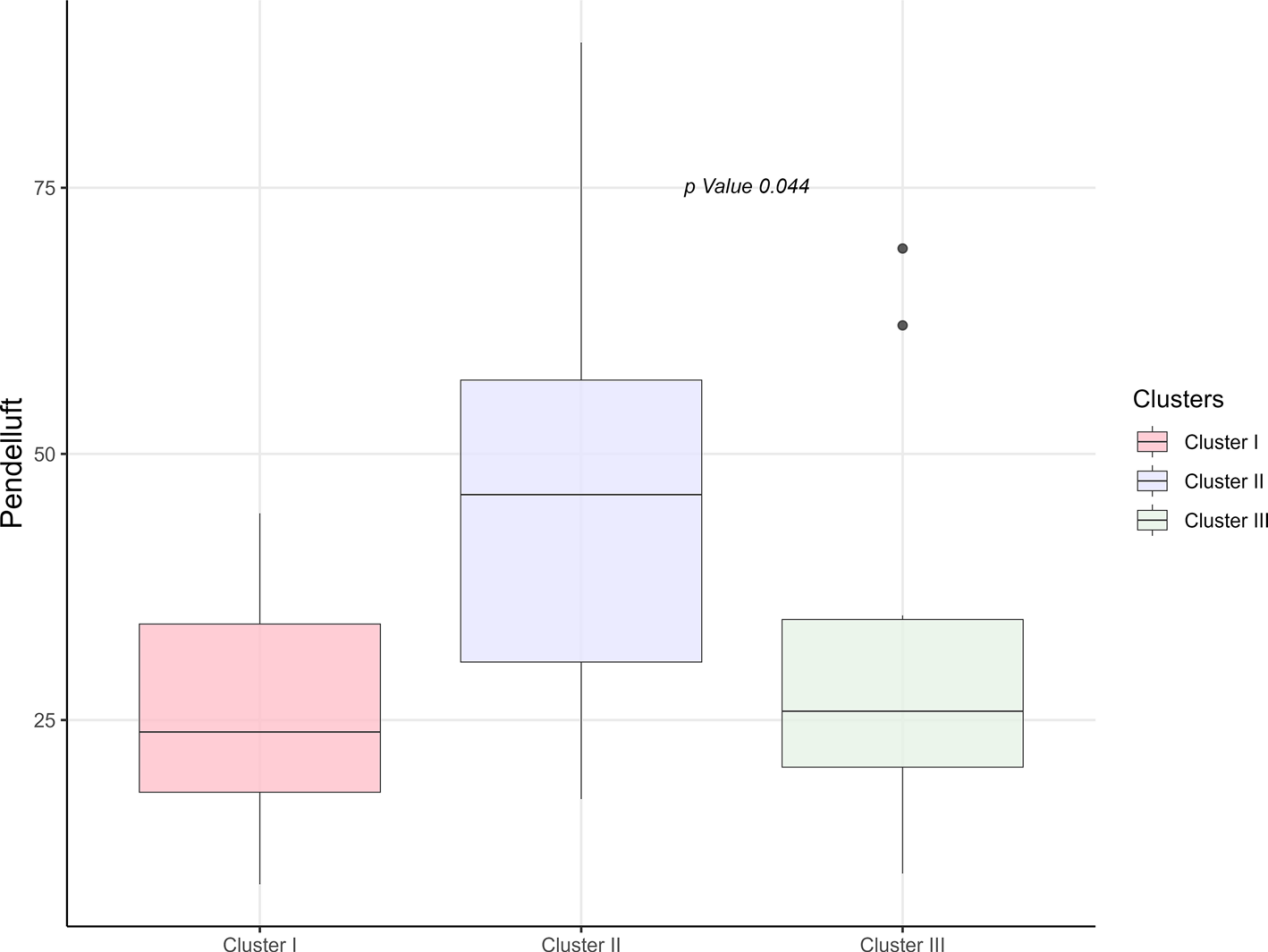
Pendelluft differences among clusters. Distribution of pendelluft (%) values across the three clusters. The figure illustrates cluster-level variability in pendelluft magnitude, reflecting differences in regional ventilation heterogeneity. Boxplots indicate line at median, interquartile range

EIT-derived parameters are reported in Table 5. Significant differences among clusters were observed for pendelluft (Fig. 4). Specifically, C2 exhibited higher pendelluft values compared with C1 and C3 (23.8 [17.9–36.1]% vs 46.2[29.8–58.1]% vs 25.8[20.3–34.6]%, *p* = 0.044). As concerns ventilation distribution, CoV-X differed significantly across clusters (*p* = 0.019). CoV-X values were lower in C1 and C2 (10.5 [7.8–12.2] vs 11.4 [5.7–13.1] vs 17.5 [11.3–18.2]), indicating a shift toward right-sided ventilation predominance in C1 and C2, whereas C3 showed values closer to symmetry (CoV-X = 16) with a mild left-sided predominance (Figure S3).

**Table 5:** EIT results in the different EIT clusters and in the global population

	Cluster I (*n* = 10)	Cluster II (*n* = 10)	Cluster III (*n* = 12)	Overall (*n* = 32)	*p* value
Pendelluft (%)	23.8 [17.9–36.1]	46.2 [29.8–58.1]	25.8 [20.3–34.6]	28.9[19.5–44.1]	**0.044**
Center of ventilation. X axis (right-to-left)	10.5 [7.8–12.2]	11.4 [5.7–13.1]	17.5 [11.3–18.2]	11.8 [7.9–16]	**0.019**
Center of ventilation. Y axis (dorsal-to-ventral)	16.8 [13.8–19.1]	15.6 [14.2–17.7]	16.3 [13.7–20.2]	15.7 [14.1–19]	0.833
Inhomogeneity Index	0.51 [0.48–0.52]	0.50 [0.49–0.52]	0.51[0.49–0.55]	0.51 [0.48–0.53]	0.646
% of TV, ROI 1	19.8 [16.2–22.6]	17.1[8.9–22.6]	14.5 [6.8–25.7]	17.7 [10.6–24.2]	0.522
% of TV, ROI 2	31.6 [24.6–51]	38.1 [24.8–47.3]	40.8 [30.2–44.7]	37.1 [27.1–46.7]	0.739
% of TV, ROI 3	28.9 [15.5–52.2]	37.3 [30.7–47.6]	43.5 [21.3–49.7]	37.3 [21.3–49.7]	0.968
% of TV, ROI 4	8.3 [3.9–12.6]	6.1 [4.9–11.4]	5.6 [2.6–11]	6.2 [3.6–12.4]	0.861
DFV (%)	38.6 [24–66.6]	48.6 [39.4–59.1]	46.6 [28.7–56.3]	44.1 [28.4–59.9]	0.977

TV: tidal volume; ROI: region of interest; DFV: dorsal fraction ventilation. Center of ventilation is based on a Cartesian axis; 0 is the most dorsal-right part of the image; 32:32 is the most ventral left part

### Comparison between low-pendelluft clusters

To further explore clinical differences between clusters characterized by lower and similar percentage of pendelluft (C1 and C3), a dedicated comparison was performed (table S1). Comorbidities, in particular cardiovascular comorbidities (9/10 (60%) vs 6 (50%)) and neurological comorbidities (3 [30%] vs 0 [0%]), were significantly more prevalent in C1. Patients in C1 were also significantly shorter than those in C3 (164.5 [160.0–168.0] vs 171.5 [166.75–178.5] cm, *p* = 0.020; online supplementary Figure S1).

### Intubation and mortality among clusters

**Fig. 5:**
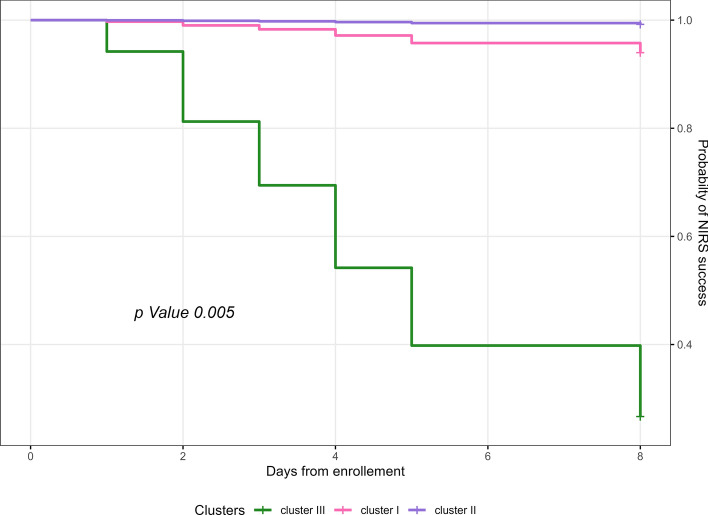
Kaplan–Meier curve displaying the results of the penalized Cox proportional hazards regression assessing the association between clinical variables and the risk of intubation. Hazard ratios with 95% confidence intervals are shown for cluster membership and selected physiological variables (ROX index, PaCO₂, age, BMI)

When evaluating the association between cluster membership and intubation within 7 days, crude intubation rates did not differ significantly among clusters (Table 6). However, in the adjusted penalized Cox regression, cluster membership was independently associated with intubation risk after adjustment for BMI, age, PaCO₂, and ROX index (Table 7, Fig. 5). Specifically, cluster C3 was associated with a higher risk of intubation.

**Table 6:** Clinical outcome in the different EIT clusters and in the global population

	Cluster I (*n* = 10)	Cluster II (*n* = 10)	Cluster III (*n* = 12)	Overall (*n* = 32)	*p* value
Intubation at 7 days	2 (20%)	1 (10%)	5 (41.7%)	8 (25%)	0.211
Alive at 7 days	9 (90%)	8 (80%)	11 (91.7%)	28 (87.5%)	0.683
Discharged at 7 days	6 (60%)	4 (40%)	7 (58.3%)	17 (53.1%)	0.603
Intubation at 28 days	2 (20%)	1 (10%)	5 (41.7%)	8 (25%)	0.211
Alive at 28 days	7 (70%)	8 (80%)	7 (58.3%)	22 (68.8%)	0.548
Discharged at 28 days	6 (60%)	7 (70%)	7 (58.3%)	23 (71.9%)	0.474
NIRS days at 7 days	2.5 [2, 3]	3 [2–5]	2 [1–3]	3 [2–4]	0.281
IMV days at 7 days	0 [0–0]	0 [0–0]	0 [0–2]	0 [0–0.5]	0.206
NIRS days at 28 days	2.5 [2, 3]	3 [2–5]	3 [1–5]	3 [1–5]	0.799
IMV days at 28 days	0 [0–0]	0 [0–0]	0 [0–4.5]	0 [0–0.5]	0.378
Ventilation-free days at 28 days	28 [0–28]	28 [17–28]	20.5 [0–28]	28 [0–28]	0.426
ICU length of stay (days)	26.5 [14–46]	29.5 [14–42]	17 [10–26]	20.5 [11–39]	0.375
Hospitalization days	26.5 [14–46]	34.5 [17–43]	20.5 [12–30.5]	23 [14.5–41.5]	0.521

IMV: invasive mechanical ventilation; NIRS: non-invasive respiratory support; ICU: Intensive Care Unit

**Table 7:** Penalized Cox regression for intubation risk assessment

	Hazard risk	Confidence interval (95%)	*p* value
Cluster membership (reference Cluster 3)			
Cluster 1	0.115	0.01–0.698	**0.017**
Cluster 2	0.042	0.002–0.35	**0.002**
ROX index	0.643	0.433–0.868	**0.001**
PaCO_2_, mmHg	1.12	1.023–1.178	**0.011**
Age, years	1.06	0.992–1.149	0.085
BMI, kg/m^2^	1.152	1.009–1.323	**0.038**

Results are reported as hazard ratios (HR) with 95% confidence intervals (95% CI) and *p* values. Membership in Cluster 1 and Cluster 2, compared with Cluster 3 (reference category), is associated with a significantly lower risk of intubation. Higher ROX Index (ratio of SpO₂/FiO₂ to respiratory rate) values are protective, whereas higher arterial partial pressure of carbon dioxide (PaCO₂) and body mass index (BMI) are associated with an increased risk of intubation. Age (years) shows a non-significant trend toward increased risk

Compared with C3, C2 showed a hazard ratio (HR) for intubation of 0.042 (95% CI 0.002–0.35, *p* = 0.002), while C1 showed an HR of 0.115 (95% CI 0.01–0.698, *p* = 0.017) (Table 7). No statistically significant differences in mortality were observed among clusters.

## Discussion

In this study, we evaluated the feasibility of using an unsupervised clustering approach applied to electrical impedance tomography (EIT) tidal images to identify physiological clusters in patients with acute hypoxemic respiratory failure (AHRF) undergoing non-invasive respiratory support (NIRS). Furthermore, association with intubation was explored as a pragmatic endpoint reflecting escalation of respiratory support, rather than as a purely negative clinical outcome. The main findings can be summarized as follows: (1) spectral clustering of raw EIT tidal images identified three distinct patient clusters; (2) these clusters differed not only in EIT-derived ventilation patterns but also in demographic, clinical, and severity characteristics; and (3) after adjustment for relevant confounders, membership to cluster 3 was independently associated with an increased risk of intubation within 7 days from NIRS initiation.

Clustering techniques have been increasingly used in critical care to uncover latent phenotypes in heterogeneous syndromes, including sepsis, ARDS, and acute respiratory failure [28]. Previous studies have applied clustering to EIT-derived features in invasively ventilated patients, mainly focusing on ventilation–perfusion matching or predefined physiological indices [27]. In contrast, the present study extends this concept to spontaneously breathing patients receiving non-invasive support and demonstrates that clustering based only on raw EIT tidal images—without a priori feature extraction—can identify distinct physiological patterns with clinical relevance.

From an EIT perspective, the clusters were primarily distinguished by differences in pendelluft magnitude and ventilation symmetry. Cluster C1 was characterized by low pendelluft and right-side ventilation, cluster C2 by high pendelluft and right-side ventilation, and cluster C3 by low pendelluft with relatively symmetric right–left ventilation. These findings suggest that EIT clustering captures not only regional ventilation distribution, but also dynamic intrapulmonary phenomena related to patient effort and lung mechanical heterogeneity.

Importantly, clusters also differed in biometric and severity-related characteristics. When comparing clusters with similarly low pendelluft (C1 and C3), patients in C1 were significantly shorter and had a higher burden of comorbidities. These differences should be interpreted as data-driven physiological hypotheses rather than definitive causal explanations. Shorter stature has previously been associated with different outcomes in mechanically ventilated ARDS patients [29] potentially reflecting anatomical and mechanical differences affecting lung–chest wall interaction. Moreover, the rightward shift of ventilation observed in C1 may reflect the mechanical influence of cardiomegaly, consistent with the higher prevalence of cardiovascular disease in this cluster and with previous observations of cardiac–pulmonary interactions affecting regional ventilation [30]. Taken together, these findings suggest that EIT-derived asymmetry may integrate structural, anthropometric, and cardiovascular determinants, generating testable hypotheses for future targeted physiological studies.

Cluster membership was also associated with clinically relevant outcomes. After adjustment for BMI, age, PaCO₂, and the ROX index, patients in cluster C3 exhibited the highest risk of intubation within 7 days. Interestingly, no difference was found among clusters in classical predictors of NIRS failure, such as the HACOR score, underlying the possible limitations of single tools in explaining intubation risk [31]. Despite lower pendelluft and a relatively symmetric ventilation pattern, Cluster 3 showed the highest intubation rate, indicating that symmetry of ventilation alone does not necessarily imply favorable respiratory mechanics [32] or clinical prognosis but can suggest, on the other side, bilateral lung involvement.

This apparent paradox highlights the complexity of NIRS failure mechanisms and the progression of Patient Self-Inflicted Lung Injury (P-SILI). Indeed, pendelluft might be a late-occurring phenomenon, and single EIT-derived parameter analysis, particularly at an early stage, may fail to capture disease progression. Furthermore, the cut-off value defining a harmful percentage of pendelluft remains unknown.

The interaction between NIRS failure and pendelluft is physiologically complex and likely linked to several factors. For instance, Cluster 3 exhibited a trend toward greater use of sedatives, which could have reduced pendelluft by lowering respiratory muscle activity, while simultaneously affecting NIRS outcome by modulating muscle effort [33, 34]. Further studies investigating the relationship between pendelluft, sedation, and intubation are warranted to better clarify this interaction.

The decision of intubating a patient with AHRF is complex, and includes progressive parenchymal disease with worsening gas exchange, excessive respiratory drive leading to patient self-inflicted lung injury (P-SILI), or clinical deterioration unrelated to ventilation distribution alone [35].

In the present dataset, the specific reasons for intubation could not be systematically determined, limiting mechanistic interpretation. Nevertheless, ventilatory therapy escalation with intubation should not always be considered a “bad” outcome per se. The observed trend toward better 7-day survival and ICU outcomes in C3, despite higher intubation rates, further supports the notion that early intubation in this group of patients may represent a protective rather than deleterious event [36]. This hypothesis, however, requires confirmation in larger, prospective studies specifically designed to evaluate timing and indications for intubation across EIT-defined phenotypes.

The heterogeneity of AHRF prevents the identification of effective, standardized respiratory and pharmacological interventions. Phenotype-driven approaches have the potential to overcome this limitation by enabling more targeted research questions and, eventually, personalized clinical strategies. In this context, EIT represents a particularly attractive tool, as it is non-invasive, bedside-available, and capable of providing continuous physiological information. A key strength, but also an interpretative challenge, lies in the comparison between clusters with similarly low pendelluft (C1 and C3). While the observed differences in stature, cardiovascular comorbidity burden, and ventilation asymmetry are physiologically plausible, they should be considered hypothesis-generating rather than confirmatory. Interestingly, although the clustering analysis was performed exclusively on EIT-derived images, without incorporating clinical variables, it was able to group patients with similar comorbidity burdens. The rightward shift in ventilation observed in Clusters 1 and 2 was consistent with their higher prevalence of comorbidities. Although derived from different domains, EIT identified specific profiles. This concordance supports the hypothesis that regional ventilation patterns may be associated with baseline clinical status. These observations provide a framework for future studies specifically designed to test these mechanistic links.

The finding that clusters with lower pendelluft were associated with a lower probability of NIRS success at seven days challenges the assumption that pendelluft and ventilation distribution alone are adequate early markers of NIRS failure risk in AHRF. The optimal predictive parameter or ventilation pattern remains yet to be identified. Nevertheless, the unsupervised clustering of raw EIT tidal images highlights a promising and hypothesis-generating approach that deserves further exploration in future EIT-based research.

Our study has some limitations. First, the sample size was relatively small (32 patients), which is acceptable for exploratory physiological research, but limits generalizability and precludes clinically usable phenotypes. Larger cohorts are needed to validate EIT images-based clustering techniques and implement phenotyping in clinical practice. Second, follow-up was limited to 28 days for ICU outcomes, with incomplete data on long-term survival and post-ICU morbidity. Third, hemodynamic data collection was limited to heart rate and arterial blood pressure; more detailed assessments of cardiac function, volume status, and right ventricular performance could have refined cluster interpretation. Additionally, direct measurements of respiratory drive and effort (e.g., esophageal pressure, diaphragmatic activity) were not available, preventing a more precise characterization of the contribution of patient effort and P-SILI. Fourth, considering the observational nature of the study, we did not protocolize criteria for intubation and therefore intubation criteria reflected local practice. This might limit generalizability of the results on intubation outcome.

Fifth, the association between EIT-derived parameters (i.e., pendelluft, ventilation symmetry) and intubation must be considered exploratory given the possibility of residual unmeasured confounding factors (e.g., effort level, lung compliance, recruitability), limited sample size and methodological limitations related to the nature of the study. Therefore, the results should be interpreted as hypothesis generating and need further confirmation by properly designed trials.

Finally, given the observational nature of the study, causality cannot be inferred, and the clinical integration of EIT-based clustering remains to be tested in properly designed trials. The clinical implication of the current results must be therefore interpreted as exploratory and requires further validation.

## Conclusions

Unsupervised clustering of EIT tidal images revealed physiological heterogeneity among patients with acute hypoxemic respiratory failure receiving non-invasive respiratory support that is not captured by conventional EIT, clinical and gas-exchange parameters. Cluster membership was independently associated with the probability of intubation within 7 days. These findings support the role of EIT-based clustering as a bedside, physiology-driven tool for early patient stratification and provide a rationale for future studies aimed at developing personalized management strategies in AHRF.

## Supplementary Information

Supplementary material 1.

## Data Availability

The data that support the findings of this study are available from the corresponding author, GS, upon reasonable request.
