# Supplementary material for: Identification of physiological clusters in acute hypoxemic respiratory failure patients undergoing non-invasive respiratory support using EIT-based t-SNE and spectral clustering
Source: Intensive Care Med Exp. 2026 May 28;14:63. doi: 10.1186/s40635-026-00912-6 (PMC13219642; doi:10.1186/s40635-026-00912-6)
Supplement: Supplementary file 1 — Supplementary material 1. [file 40635_2026_912_MOESM1_ESM.docx]

**Identification of physiological clusters of acute hypoxemic respiratory failure patients undergoing non-invasive respiratory support using EIT-based t-SNE and spectral clustering**

Gaetano Scaramuzzo, Valentina Bellini, Matteo Trevisani, Francesca Cinquegrana, Marta Bonanni, Marta Ciniero, Matteo Riccardo, Pierluigi Ferrara, Alessandro Trentini, Tiziana Bellini, Giulia Tini, Sara Uboldi, Danila Azzolina, Savino Spadaro, Carlo Alberto Volta, Elena Giovanna Bignami

**ONLINE DIGITAL SUPPLEMENT**

**Additional Methods**

**Formulas for EIT derived parameters**

1. **Pendelluft** was calculated using the impedance-based method proposed by Menga et al.[7]

$pendelluft\%=\frac{\Sigma_{i=N}(Pixel\_end\_inspZ_{pixel}-Pixel\_end\_expZ_{pixel})-\Sigma_{i=N}(Global\_end\_inspZ_{pixel}-Global\_end\_expZ_{pixel})}{\Sigma_{i=N}(Pixel\_end\_inspZ_{pixel}-Pixel\_end\_expZ_{pixel})}$ *100

1. **Regional ventilation distribution across four regions of interest** (ROI 1: most ventral to ROI 4: most dorsal, defined as fixed regions of 8 pixel rows each) was calculated as follows:

$${ROI\left( k \right)}_{ventilation\%}=\frac{\Sigma\Delta Z\_i(ROI(k))}{\Sigma\Delta Z\_i(Global)}$$

2. **Center of ventilation (CoV),** defined as the geometric mean of the tidal impedance variation and characterized by two variables, x (horizontal axis, 0=right; 32=left) and y (vertical axis, 0=dorsal, 32 =ventral) [22] was calculated using the [regionprops](https://it.mathworks.com/help/matlab/ref/polyshape.centroid.html#d126e1391338) function in Matlab R2025a.
3. The **global inhomogeneity index**, defined as the inhomogeneity among different pixel’s relative impedance variation [22] was calculated as follows:
   $GI=\frac{\Sigma_{x,y\in lung}|{\Delta Z}_{xy}-median{\Delta Z}_{lung}|}{\sum_{x,y\in lung} {\Delta Z}_{xy}}$
   - - $\Delta Z$*xy is the pixel in the identified lung area;*
     - $\Delta Z$*lung is all the pixels representing the lung area.*
4. **The dorsal fraction of ventilation (DFV)** was defined as the percentage of tidal volume received by the dorsal part of the lung and calculated as follows:

DFV= (ROI3%+ROI4%) / (ROI1%+ROI2%)

**Formulas for image analysis (t-SNE)**

$normalization of pixels[0,1]=\frac{(X - min(X))}{(max(X) - min(X))}$

$CosineSimilarity(X,Y)=\frac{X x Y}{||X|| ||Y||}$

$CosineDistance(X,Y)=1-CosineSimilarity(X,Y)$

**Additional tables**

*Tab S1. Demographic features comparison in low pendelluft clusters*

|  | **Cluster I**  **(n=10)** | **Cluster III**  **(n=12)** | **p Value** |
| --- | --- | --- | --- |
| **Age (years)** | 71[66-77] | 65[57.5-75] | 0.30 |
| **Sex, female** | 5(50%) | 4(33%) | 0.429 |
| **Height (cm)** | 164.5 [160–168] | 171.5 [167–179] | **0.02** |
| **BMI (kg/m2)** | 27.3[23.9-31.1] | 25.5[23.8-28.3] | 0.57 |
| **SOFA score** | 4.5[2-6] | 3[2-4.5] | 0.5 |
| **SAPS II** | 50[45-67] | 42.50 [37–49.75] | 0.08 |
| **ROX Index** | 10.2[5.8-11.1] | 8.9[6.9-9.9] | 0.48 |
| **Murray Score** | 1.5[1-1.5] | 2[1.5-2.5] | 0.073 |
| **Total fluid balance (ml/kg)** | 38.5 [18–82] | 37.7 [4.7–139] | 0.87 |
| **Comorbidities** |  |  |  |
| *Chronic Kidney disease* | 1(10%) | 1(8.3%) | 0.892 |
| *Respiratory disease* | 4(40%) | 1(8.3%) | 0.078 |
| *Neurological disease* | 3(30%) | 0(0%) | **0.041** |
| *Cardiovascular disease* | 9(90%) | 6(50%) | **0.045** |
| *Metabolic disease* | 4(40%) | 4(33%) | 0.746 |
| **Non-smoker** | 8(80%) | 12(100%) | 0.078 |
| **Ex-smoker** | 1(10%) | 0(0%) |
| **Smoker** | 1(10%) | 0(0%) |
| **Admission diagnosis** |  |  |  |
| *Pneumonia* | 2(20%) | 3(25%) | 0.729 |
| *Shock* | 5(50%) | 4(33%) | 0.429 |
| *Neuromuscular diagnosis* | 2(20%) | 0(0%) | 0.104 |
| *Post-surgery admission* | 1(10%) | 5(41.6%) | 0.097 |
| *Trauma* | 0(0%) | 0(0%) | 1 |
| **Support modality** |  |  |  |
| *NIV* | 3(30%) | 4(33%) | 0.867 |
| *HFNC* | 7(70%) | 8(67%) |

BMI, Body Mass Index. SOFA, Sequential Organ Failure Assessment. SAPS II, Simplified Acute Physiology Score II. NIV, Non-Invasive Ventilation. HFNC, High Flow Nasal Cannula.

*Tab S2. Clinical parameters comparison in low-pendelluft’s clusters*

|  | **Cluster I**  **(n=10)** | **Cluster III**  **(n=12)** | **p Value** |
| --- | --- | --- | --- |
| **Mean blood pressure (mmHg)** | 89.2[70-93.3] | 86.6 [82.2-96] | 0.59 |
| **Systolic blood pressure (mmHg)** | 117 [101–136] | 136 [120–140] | 0.207 |
| **Heart rate (bpm)** | 83[74-110] | 94[73.5-109] | 0.70 |
| **Shock Index** | 1.34[0.99-1.87] | 1.45[1.25-1.79] | 0.242 |
| **Oxygen Saturation (%)** | 97[95-98] | 97.5[96-98.5] | 0.94 |
| **Respiratory rate (breaths/min)** | 20.5[17-30] | 22.5[17-28] | 0.84 |
| **FiO2 (%)** | 53.5 [44.7-59] | 50[44-58.7] | 1 |
| **PaO2/FiO2 (mmHg)** | 185[130-255] | 173[120.5-222] | 0.41 |
| **Adjunctive Therapy** |  |  |  |
| *Sedatives, yes* | 6(60%) | 10(83%) | 0.221 |
| *Corticosteroids, yes* | 5(50%) | 5(41.6%) | 0.696 |
| *Vasoactive, yes* | 3(30%) | 2(16%) | 0.49 |
| *Acute antihypertensives use, yes* | 4(40%) | 8(67%) | 0.211 |

FiO2, Fraction of inspired oxygen. PaO2, oxygen arterial partial pressure.

*Tab S3. Laboratory findings comparison in low-pendelluft clusters*

|  | **Cluster I**  **(n=10)** | **Cluster III**  **(n=12)** | **p Value** |
| --- | --- | --- | --- |
| **pH** | 7.45[7.43-7.52] | 7.46[7.40-7.48] | 0.84 |
| **PaO2 (mmHg)** | 92.2[75.8-101] | 87.9[73.7-100] | 0.84 |
| **PaCO2 (mmHg)** | 42[38.2-46] | 39.8[37.6-43.9] | 0.30 |
| **Lactates (mmol/L)** | 0.9[0.7-1.2] | 1.05[0.85-1.4] | 0.68 |
| **Bicarbonates (mmol/L)** | 29.3[23.5-34.6] | 30.5[24.5-31.9] | 1 |
| **Hemoglobin (g/dL)** | 9[9-11] | 9.5[8-12.5] | 0.57 |
| **Leucocyte (x 10^3^/mm^3^)** | 10.8[7.3-15.4] | 16.6[10.9-24.6] | 0.24 |
| **Creatinine (mg/dL)** | 0.97[0.78-1.19] | 1.09[0.7-1.6] | 0.59 |
| **C-Reactive Proteine (mg/dL)** | 11.5[9-20.2] | 16.3[5-20.6] | 0.81 |
| **Procalcitonin (ng/mL)** | 2.05[0-6] | 1[0.1-2.5] | 0.61 |

PaO2: arterial Oxygen Partial Pressure. PaCO2. arterial Carbon Dioxide Partial Pressure.

*Tab S4. EIT parameters comparison of low-pendelluft clusters*

|  | **Cluster I**  **(n=10)** | **Cluster III**  **(n=12)** | **p Value** |
| --- | --- | --- | --- |
| **Pendelluft** | 23.8 [17.9-36.1] | 25.8 [20.3-34.6] | 0.53 |
| **Centre of Ventilation. X axis (right-to-left)** | 10.5 [7.8-12.2] | 17.5 [11.3-18.2] | **0.05** |
| **Centre of Ventilation. Y axis (dorsal-to-ventral)** | 16.8[13.8-19.1] | 16.3[13.7-20.2] | 1 |
| **Inhomogeneity Index** | 0.51[0.48-0.52] | 0.51[0.49-0.55] | 0.34 |
| **% of TV, ROI 1** | 19.8[16.2-22.6] | 14.5 [6.8-25.7] | 0.31 |
| **% of TV, ROI 2** | 31.6[24.6-51] | 40.8[30.2-44.7] | 0.49 |
| **% of TV, ROI 3** | 28.9[15.5-52.2] | 43.5[21.3-49.7] | 0.82 |
| **% of TV, ROI 4** | 8.3 [3.9-12.6] | 5.6[2.6-11] | 0.72 |
| **DFV (%)** | 38.6[24-66.6] | 46.6[28.7-56.3] | 0.87 |

TV, Tidal Volume. ROI, Region of Interest. DFV, Dorsal Fraction Ventilation

*Table S5. Silhouette analysis’ results.*

| **N. clusters** | **Silhouette score** | **% sample with silhouette<0** | **Cluster sizes** |
| --- | --- | --- | --- |
| **2** | 0.359 | 9.38% | [22,10] |
| **3** | 0.314 | 3.13% | [12,10,10] |
| **4** | 0.308 | 6.25% | [10, 8, 7, 7] |
| **5** | 0.317 | 6.25% | [10, 7, 5, 5, 5] |

***Table S6: Penalized Cox regression with sedation parameter for intubation risk assessment***

|  | ***Hazard Risk*** | ***Confidence Interval***  ***(95%)*** | ***p Value*** |
| --- | --- | --- | --- |
| ***Cluster membership (reference Cluster 3)*** |  |  |  |
| - *Cluster 1* | *0.110* | *0.005-0.76* | ***0.022*** |
| - *Cluster 2* | *0.042* | *0.002 – 0.37* | ***0.002*** |
| ***ROX Index*** | *0.65* | *0.44 - 0.88* | ***0.002*** |
| ***PaCO_2_, mmHg*** | *1.07* | *1.008 - 1.16* | ***0.028*** |
| ***Age, Years*** | *1.06* | *0.99 - 1.14* | *0.097* |
| ***BMI, Kg/m^2^*** | *1.152* | *0.97 - 1.30* | *0.097* |
| ***Sedation, yes*** | *0.55* | *0.078-4.59* | *0.54* |

**Figure S1. Different height in the clusters**


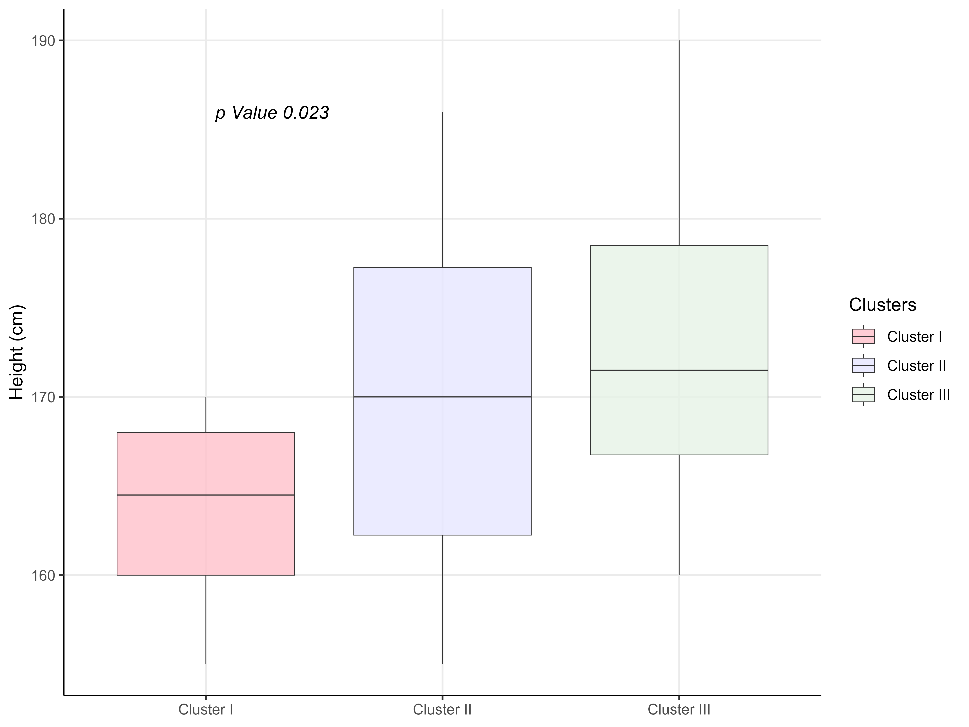


Comparison of patient height across the three identified clusters. The figure illustrates the distribution within each cluster, highlighting significant inter-cluster variability.

**Figure S2. Differences in clinical variables at baseline among clusters**


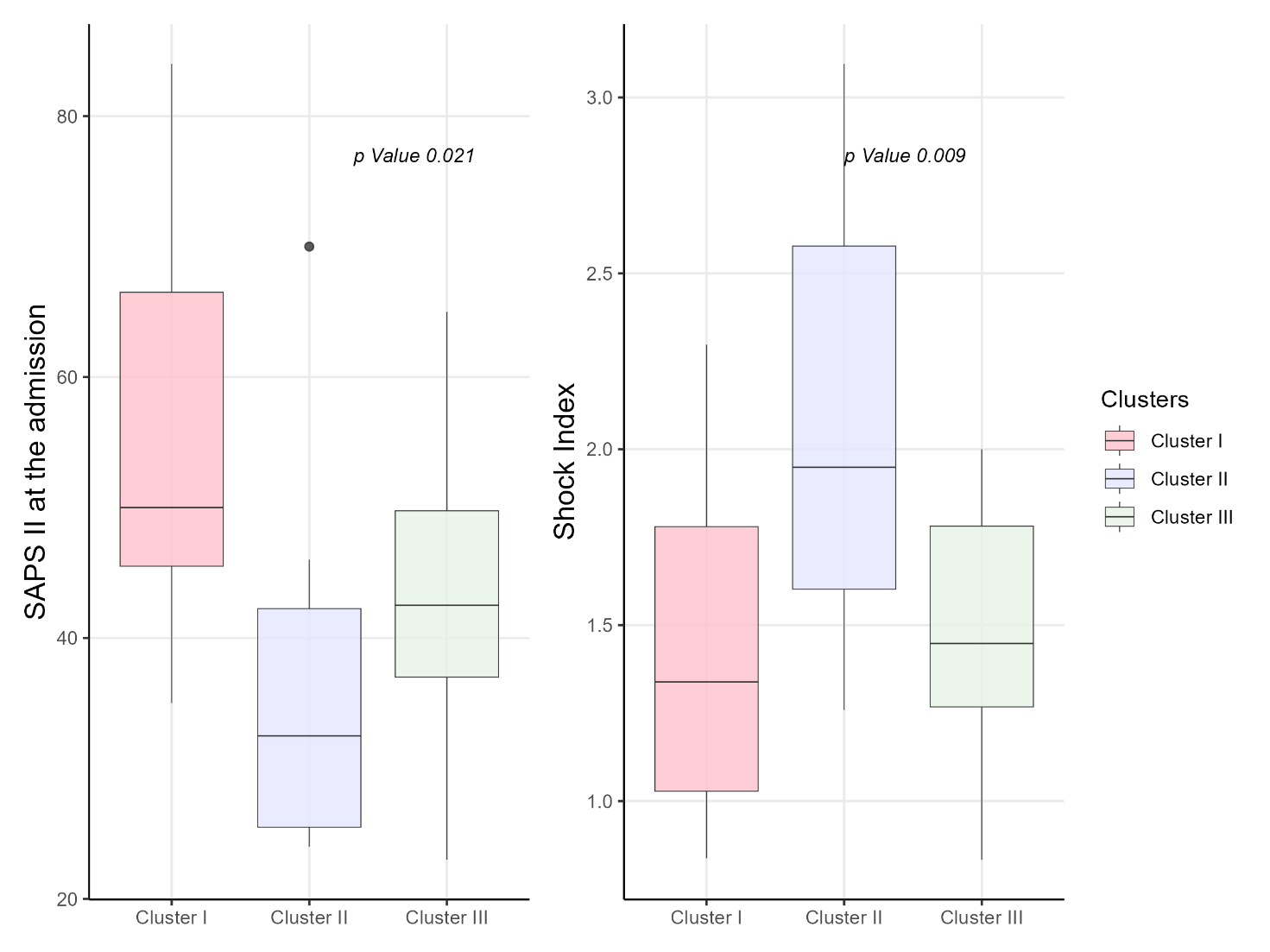


Comparative visualization of SAPS II and Shock Index across the three clusters. The figure depicts the distribution of SAPS II and Shock Index at enrollment, highlighting inter-cluster variability.

**Figure S3: Different centre of ventilation on X Axis among clusters**


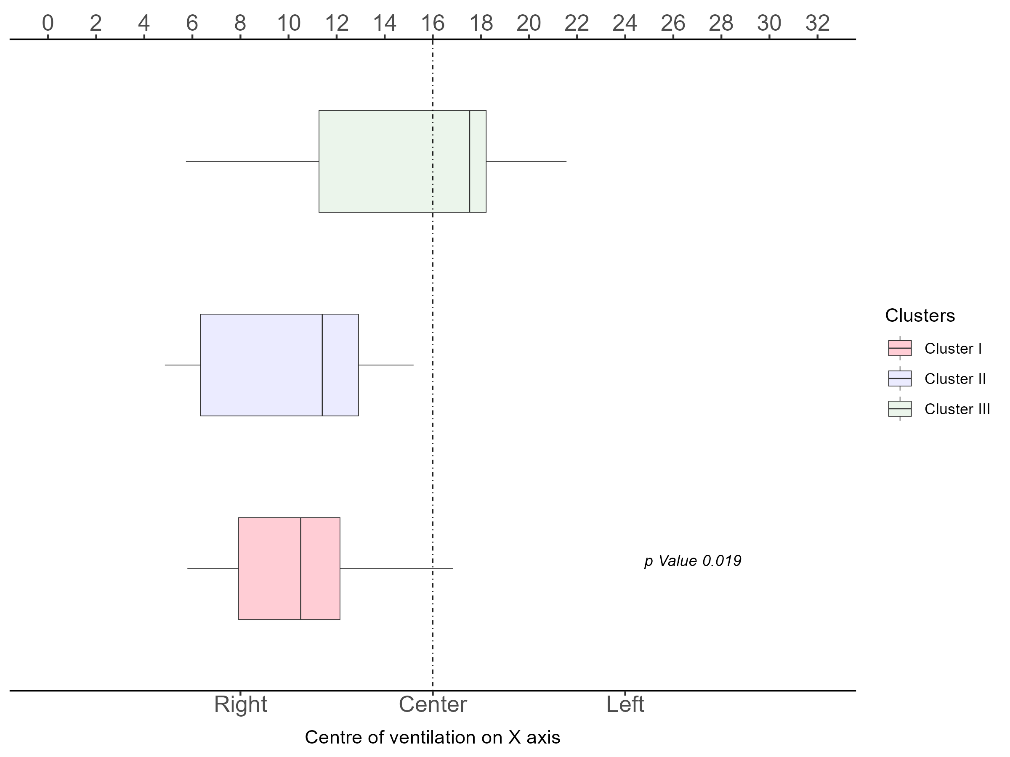


Comparative analysis of the centre of ventilation (CoV) on the X-axis, derived from electrical impedance tomography, across the three clusters. The figure shows how the distribution of lateral-to-lateral ventilation shifts between clusters, emphasizing heterogeneity in ventilation patterns and mechanical behaviour of the respiratory system.

**Figure S4: Silhouette analysis for clustering definition**


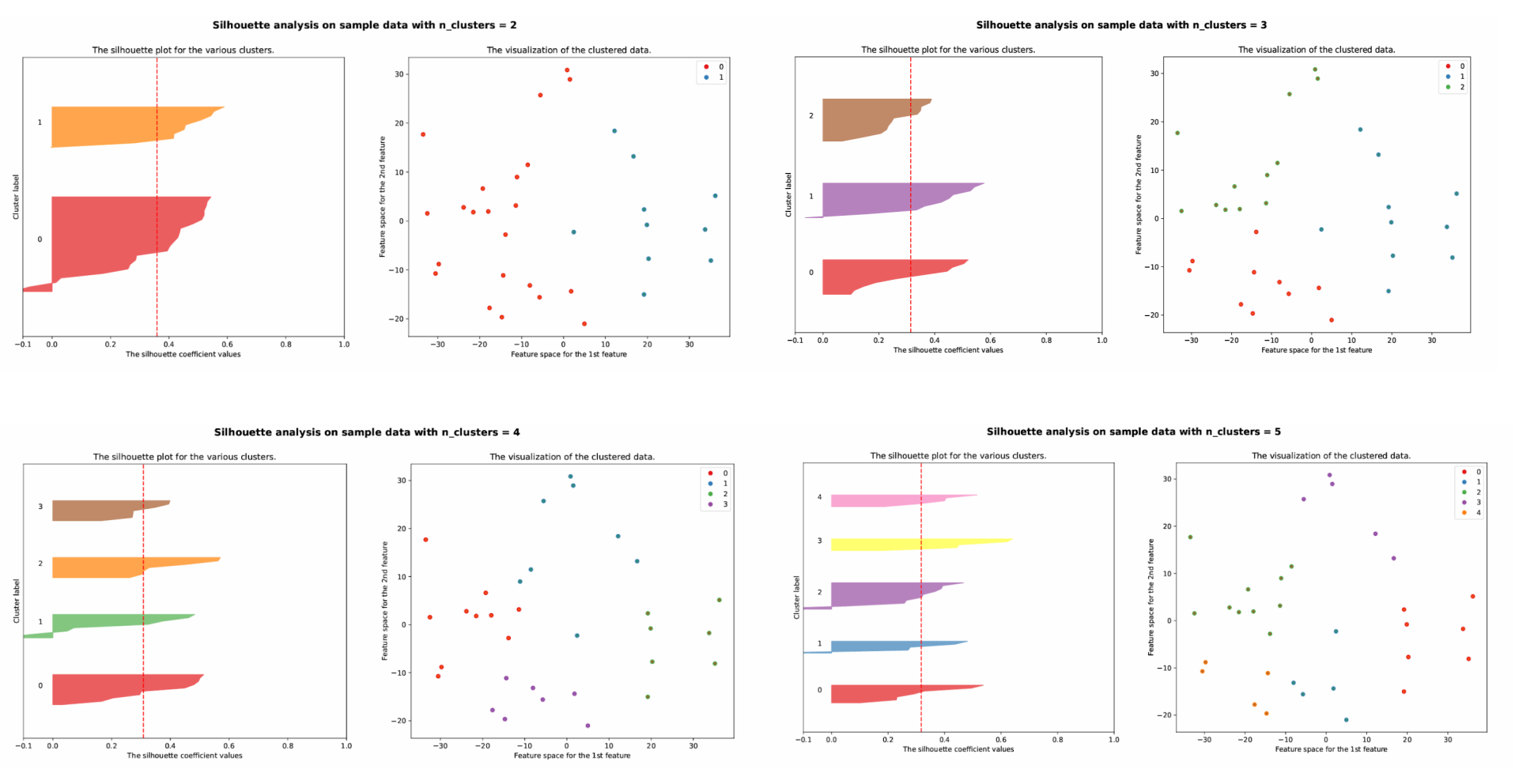


**Figure S5: Percentage of Pendelluft based on sedation status**


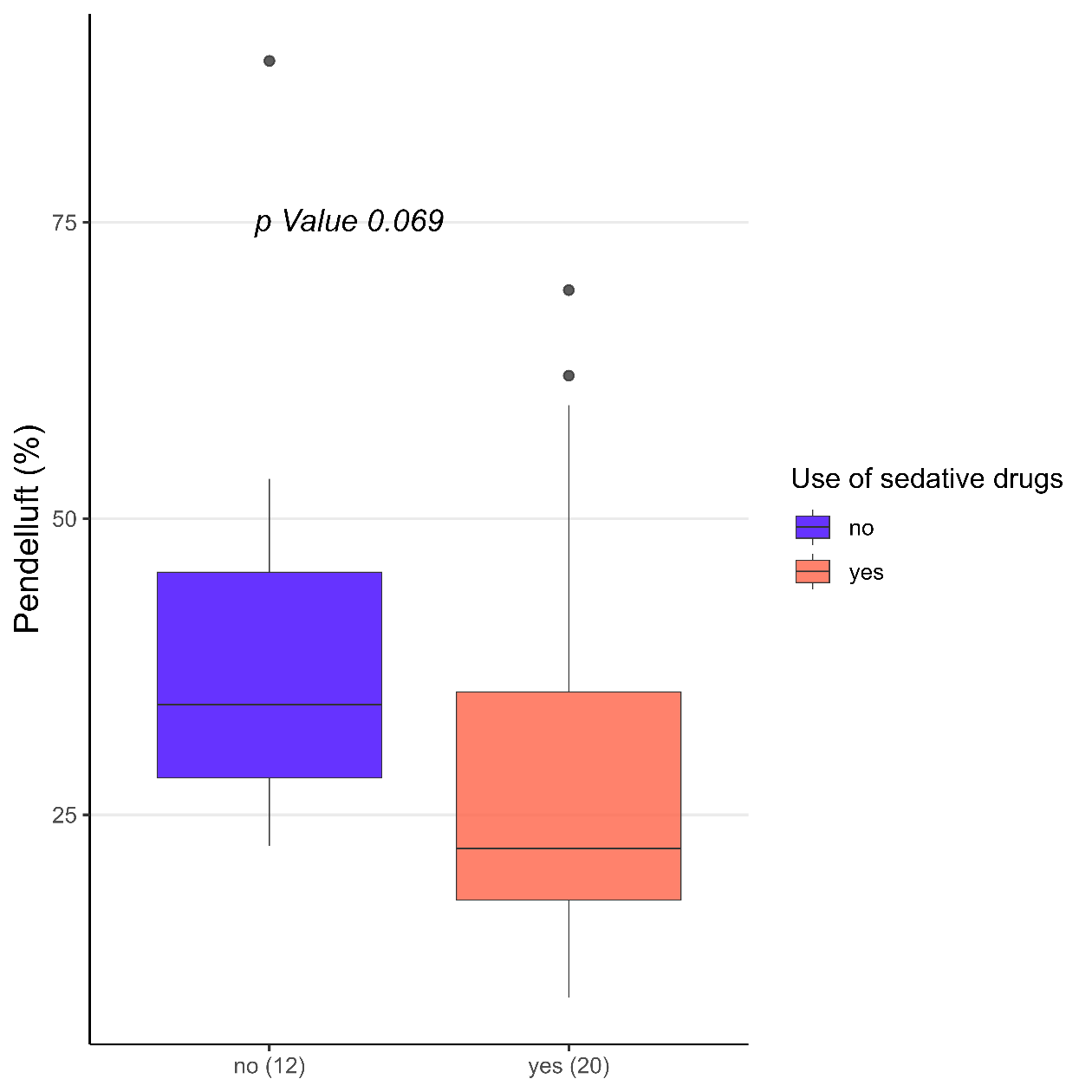


Percentage of pendelluft in the two groups of patients divided by use of sedative drugs.
